# A genome-scale CRISPR/Cas9 knockout screening reveals *SH3D21* as a sensitizer for gemcitabine

**DOI:** 10.1038/s41598-019-55893-2

**Published:** 2019-12-16

**Authors:** Mohammad Masoudi, Motoaki Seki, Razieh Yazdanparast, Nozomu Yachie, Hiroyuki Aburatani

**Affiliations:** 1 Molecular Biology Department, Graduate School of Medicine, The University of Tokyo, Tokyo, 153-8904 Japan; 2 Genome Science Division, Research Center for Advance Science and Technology, The University of Tokyo, Tokyo, 153-8904 Japan; 3 Synthetic Biology Division, Research Center for Advance Science and Technology, The University of Tokyo, Tokyo, 153-8904 Japan; 4 Molecular Biology Laboratory, Institute of Biochemistry and Biophysics, University of Tehran, Tehran, 13145-1384 Iran

**Keywords:** Cancer genomics, High-throughput screening, Pancreatic cancer

## Abstract

Gemcitabine, 2′,2′-difluoro-2′-deoxycytidine, is used as a pro-drug in treatment of variety of solid tumour cancers including pancreatic cancer. After intake, gemcitabine is transferred to the cells by the membrane nucleoside transporter proteins. Once inside the cells, it is converted to gemcitabine triphosphate followed by incorporation into DNA chains where it causes inhibition of DNA replication and thereby cell cycle arrest and apoptosis. Currently gemcitabine is the standard drug for treatment of pancreatic cancer and despite its widespread use its effect is moderate. In this study, we performed a genome-scale CRISPR/Cas9 knockout screening on pancreatic cancer cell line Panc1 to explore the genes that are important for gemcitabine efficacy. We found *SH3D21* as a novel gemcitabine sensitizer implying it may act as a therapeutic target for improvement of gemcitabine efficacy in treatment of pancreatic cancer.

## Introduction

Gemcitabine, 2′,2′-difluoro-2′-deoxycytidine (dFdC), is an analogue of deoxycytidine with two fluorine atoms, which is widely used in chemotherapy of solid tumour cancers including pancreas, bladder and breast cancers^1^. Gemcitabine is administered as a pro-drug and the only reported mechanism for its cellular uptake is transportation by human nucleoside transporter proteins SLC28A1, SLC28A3, SLC29A1 and SLC29A2^1^. Once inside the cell, gemcitabine is phosphorylated to gemcitabine mono-, di- and tri-phosphate by deoxycytidine kinase, cytidine/uridine monophosphate kinase 1 and nucleoside diphosphate kinase, respectively. Subsequently, gemcitabine triphosphate is incorporated into the DNA chain by DNA polymerases and causes “masked chain termination” of DNA replication. In this mechanism, DNA replication is halted following incorporation of one base after gemcitabine^2^ and thereby triggers cell cycle arrest and apoptosis. Furthermore, gemcitabine diphosphate influences DNA synthesis by reducing dNTP pool of the cells via covalent binding to the active site of ribonucleotide reductase and its consequent inactivation^3^. These distinct modes of action have made gemcitabine one of the most prevalent drugs for cancer treatment.

Pancreatic cancer is known as the “most lethal common cancer” with a five year overall survival rate of less than 5%^4^. More than 90% of the patients diagnosed with pancreatic cancer die and median overall survival for the patients is 8 to 12 months for locally advanced disease^5^. Chemotherapy is the first line standard treatment for unresectable pancreatic cancer and gemcitabine possesses a central role in this treatment. Although gemcitabine has been extensively administered for treatment of pancreatic cancer its efficacy is still modest, 5.6 months overall survival^6^, which warrants exploration to discover candidates for combinatorial therapy.

A variety of methods and tools are used to seek the candidates for combinatorial therapy with a certain drug. RNA-guided clustered regularly interspaced short palindromic repeats (CRISPR)/Cas nuclease system is one of the most powerful tools currently in use for screening in human cells. This system was originally discovered as a bacterial immune system that targets and cleaves foreign genomic elements in bacteria and as a consequence inactivates the invasive DNA. Later, CRISPR/Cas system was engineered and exploited to target genomic regions of interest in other species including human. Since then, it has been proved as a powerful genome editing tool^7^ which has been used in human cancer cell lines to explore the genes involved in drug resistance and essential genes for the survival of the cells^8,9^.

In this study, we utilized a genome-scale CRISPR-Cas9 knockout sgRNA library to identify modulators of gemcitabine action in Panc1 pancreatic carcinoma cell line. It has been demonstrated that Panc1 has a higher resistance to gemcitabine compared to other pancreatic cancer cell lines MIA-PaCa-2 and BxPC-3^10^. We found that *SH3D21* acts as a gemcitabine sensitizer and endocytosis is involved in gemcitabine cellular uptake. In addition, the list of essential gene sets for the survival of Panc1 cells was acquired.

## Results

### Genome-scale knockout experiment

**Figure 1:**
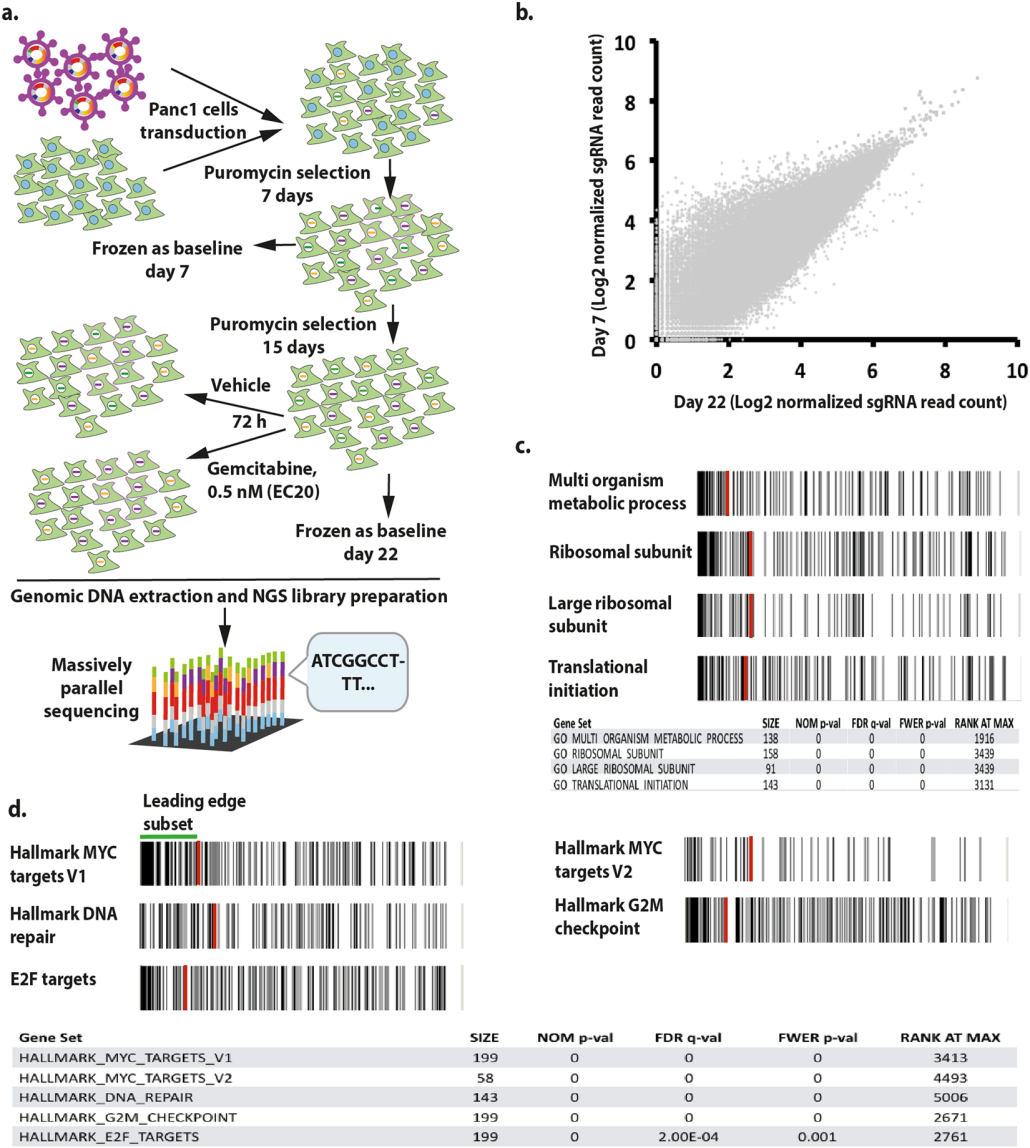
Essential gene sets for Panc1 cells survival. (**a**) Overview of the screening. (**b**) Comparing the read counts of the sgRNAs from day 7 and day 22 baseline samples. (**c**) Gene set enrichment analysis using sgRNAs’ read count of day 22 and day 7 baseline samples. Gene Ontology (GO) All gene sets were employed for the analysis with minimum size of 20 and maximum size of 200 for gene sets. (**d**) GSEA using Hallmark All gene sets with minimum size of 20 and maximum size of 200 employing sgRNAs’ read count from day 22 and day 7 baseline samples. Red line indicates rank at max. Left to the red line is leading subset.

A genome-scale knockout experiment was performed on Panc1 cells employing Genome-Scale CRISPR Knock-Out (GeCKO) version 2 sgRNA library^11^. This library targets 19,050 human genes using 123,411 unique sgRNAs (Fig. 1a). GeCKO-v2 library is composed of two sub-libraries, A and B, which their acquired coverage after massively parallel sequencing were 99.4 and 99.5%, respectively (Supplementary Fig. S2). The obtained number of the sgRNAs from both libraries were combined in further analysis and the efficacy of the genome-scale knockout experiment in Panc1 cells was assessed by comparing cells from day 7 and 22 after start of puromycin selection (Fig. 1b,c). Gene set enrichment analysis (GSEA)^12^ revealed that sgRNAs targeting essential gene sets for the survival of the cell (including Multi Organism Metabolic Process, Ribosomal Subunit and Translational Initiation) were depleted in the cells from day 22 (Fig. 1c).

### MYC pathway is essential for Panc1 cells survival

Essential gene sets for the survival of Panc1 cells were obtained by GSEA employing Hallmark All gene sets. Comparing baseline samples (drug-untreated) from day 7 and 22, it became evident that Hallmark gene sets MYC-targets, DNA-repair, G2M-checkpoint and E2F-targets act as top essential pathways for Panc1 cells survival (Fig. 1d).

### Gemcitabine screening revealed *SH3D21* as a gemcitabine sensitizer

**Figure 2:**
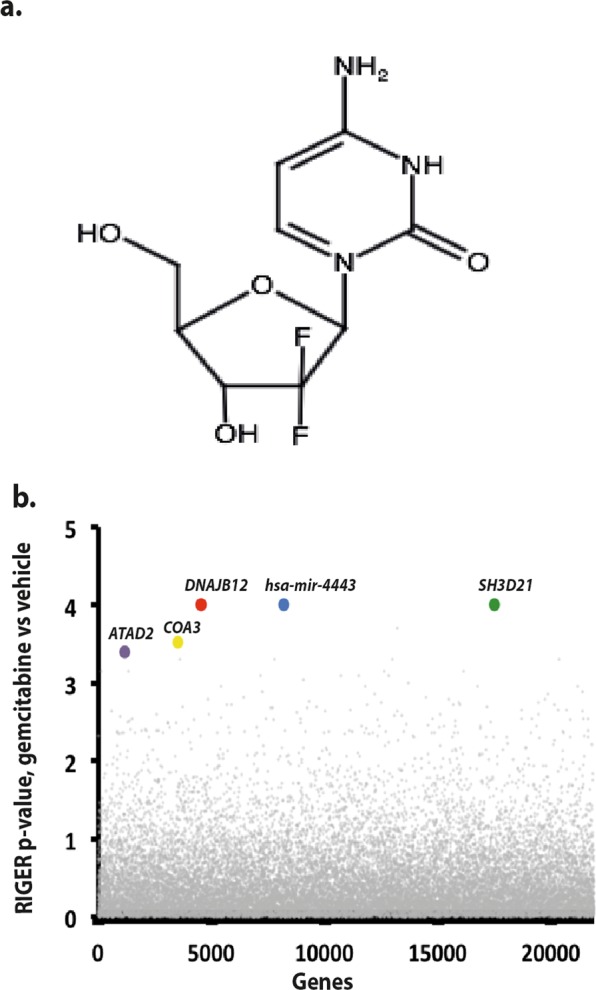
Gemcitabine and top depleted genes. (**a**) Gemcitabine, 2′,2′-difluoro-2′-deoxycytidine (dFdC). (**b**) RIGER p-value position of the top five depleted genes.

**Figure 3:**
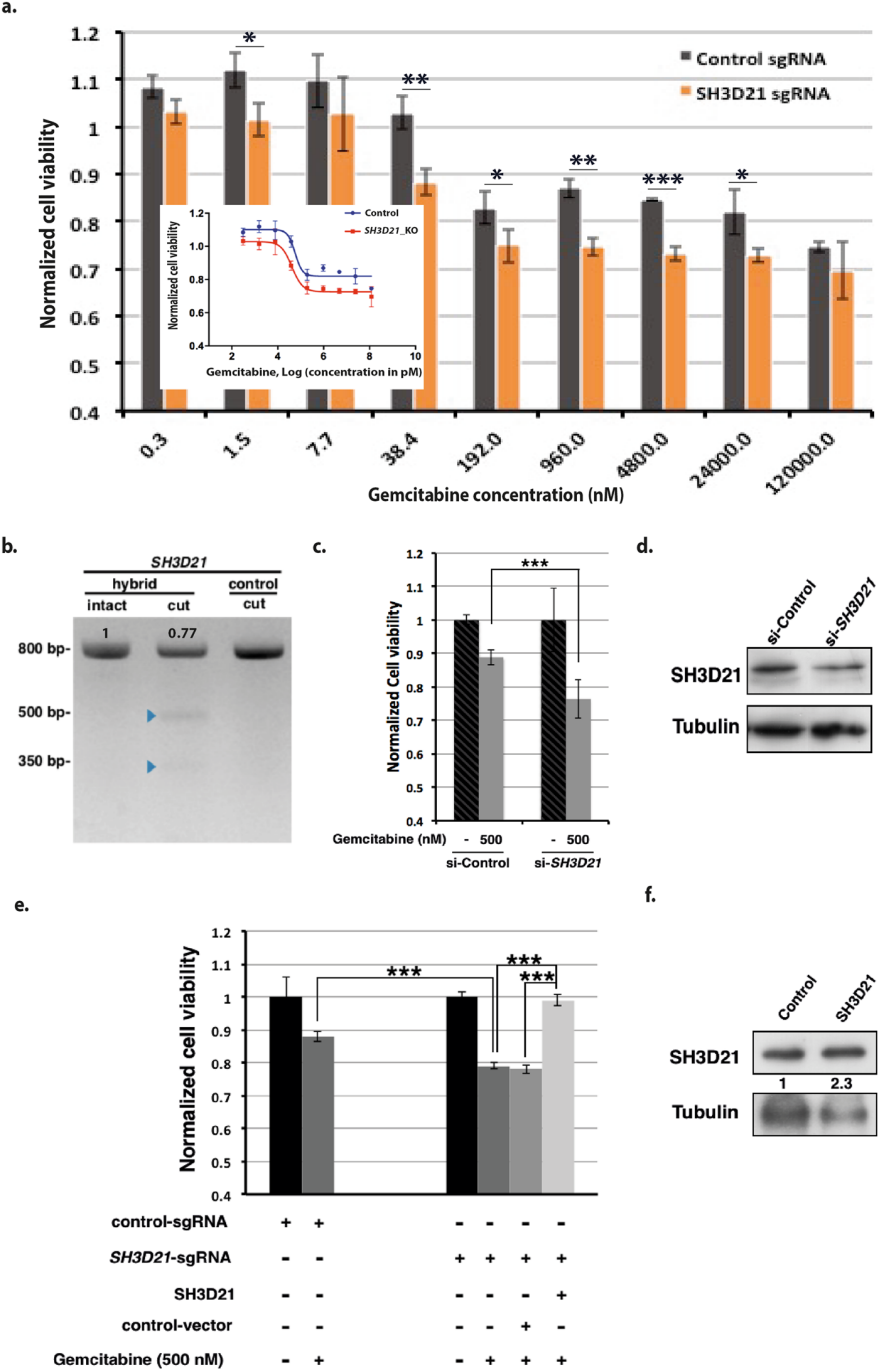
*SH3D21* is a gemcitabine sensitizer. (**a**) Cell viability assay of Panc1 cells carrying sgRNA targeting *SH3D21*. Cell viability was assessed 72 h after start of the gemcitabine treatment. Control sgRNA data is the average of two control sgRNAs. Bars show the average of three experimental replicates ± SD. * and ** represent p-values ≤ 0.05 and ≤0.001, respectively. (Inset) *SH3D21* knockout decreases the EC50 of gemcitabine in Panc1 cells, 41.1 nM in *SH3D21*-knockout cells vs 56.8 nM in control cells. (**b**) SURVEYOR assay of *SH3D21* sgRNA. Genomic region containing the sgRNA target site was PCR-amplified, 800 bp. Control and sample amplicons were mixed and reannealed. The annealing product was digested by T7 endonuclease enzyme. Indel mutations of *SH3D21* produce bands with the size of 480 and 320 bp. (**c**) si-*SH3D21* increases sensitivity of Panc1 cells to gemcitabine. (**d**) Western blot of SH3D21 protein after treatment of Panc1 cells with the si-*SH3D21*. Panels are cropped images of the same blot stained with different antibodies. Full-length blots are presented in Supplementary Fig. S3. (**e**) Re-expression of SH3D21 in *SH3D21*-knockout cells rescued the effect of *SH3D21* knockout. *SH3D21*-knockout cells were seeded and transfected with either SH3D21 expression vector or empty vector. After 24 hours the medium was change to the medium containing gemcitabine and cell viability was measured after 72 hours. (**f**) Western blot of SH3D21 re-expression in *SH3D21*-knockout cells.

Following 22 days of puromycin selection, library of Panc1 cells carrying sgRNAs was divided and subjected to either gemcitabine (Fig. 2a) (0.5 nM) or vehicle for 72 hours. After the drug screening, copy number of the sgRNAs were extracted from the genomic DNA utilizing massively parallel sequencing (Fig. 1a). RIGER (RNAi gene enrichment ranking)^13^ algorithm was used to rank the genes based on their differential effect in vehicle- and gemcitabine-treated cells. Weighted sum method of RIGER algorithm first ranks all sgRNAs based on their differential effects in gemcitabine- and vehicle-treated cells and then ranks the genes based on the position of their top two sgRNAs. *SH3D21* gene appeared as top gemcitabine sensitizer in RIGER ranked list (Table S1 and Fig. 2b). To validate the sensitizer activity of *SH3D21*, Panc1 cells bearing *SH3D21* sgRNA were subjected to cell viability assay in the presence of gemcitabine (Fig. 3a). The results indicated that gemcitabine EC50 decreased to 41.1 nM compared to that of the control cells (56.8 nM) (Fig. 3a, inset). The activity of *SH3D21* targeting sgRNA on its target site in Panc1 cells’ genome was confirmed by SURVEYOR assay (Fig. 3b). siRNA knockdown of *SH3D21* mRNA resulted in higher sensitivity of Panc1 cells to gemcitabine (Fig. 3c,d). In addition, re-expression of SH3D21 in *SH3D21*-knockout cells rescued the sensitizing effect of *SH3D21* knockout (Fig. 3e,f).

Performing RIGER algorithm, the list of enriched genes in gemcitabine-treated cells was obtained as well (Table S2). Supplementary Figure S5 shows the position of the top five enriched genes based on their RIGER p-value. Position of the top five depleted and enriched genes sgRNAs among top 1000 ranked sgRNAs are shown in Supplementary Figs. S6 and S7.

Based on their role in gemcitabine pathway^1^, the genes helping gemcitabine efficacy were categorized as positive modulators and the ones attenuating gemcitabine effect were categorized as negative modulators. Employing gene set enrichment analysis, positive modulators of gemcitabine showed enrichment in the gemcitabine-treated cells, compared to the vehicle-treated cells, while negative modulators did not show enrichment (Supplementary Fig. S8).

### PANTHER analysis and GSEA of gemcitabine screening

**Figure 4:**
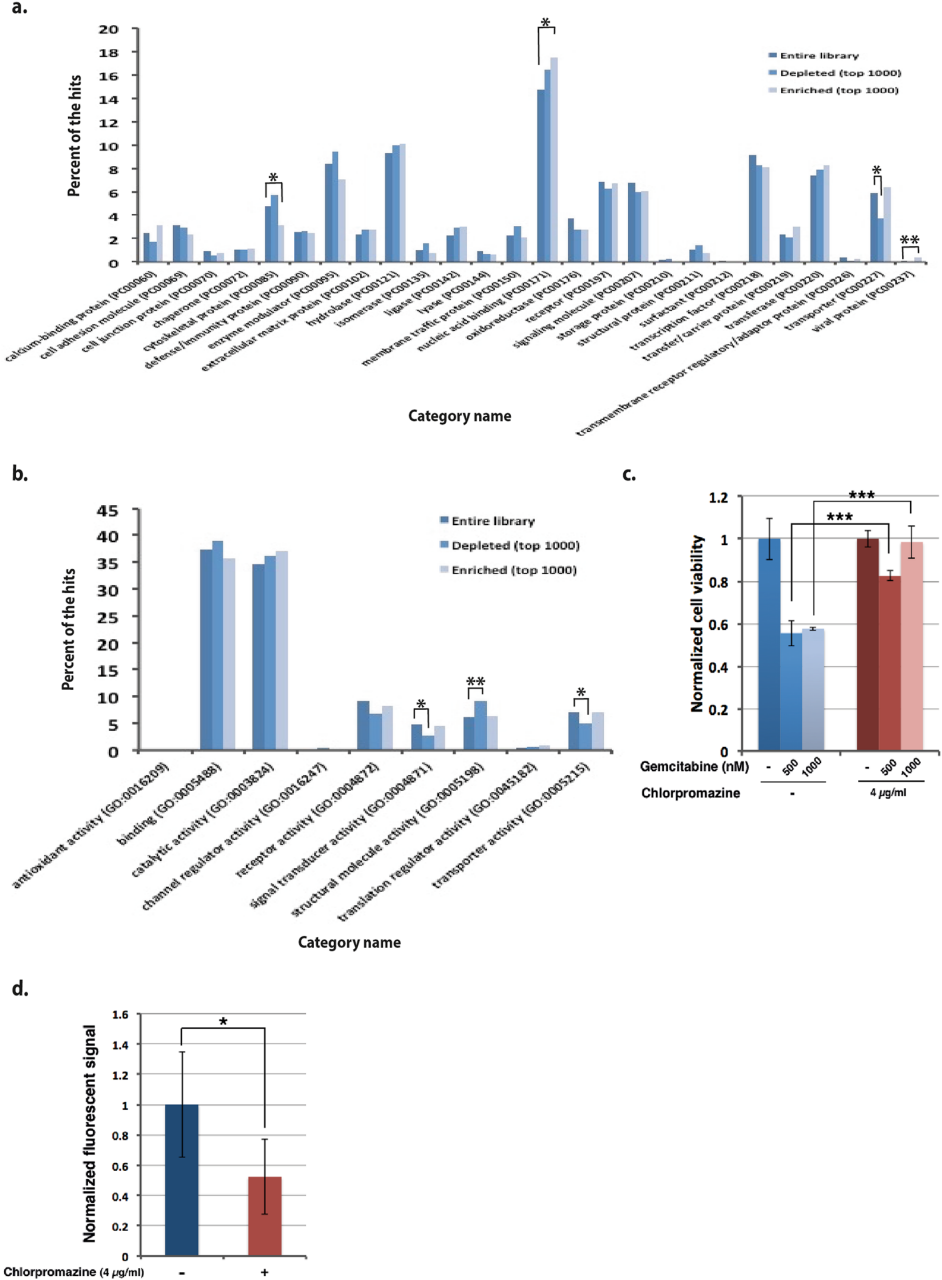
Endocytosis is involved in gemcitabine cellular uptake. Gene composition comparison of the entire library, top 1000 depleted genes and top 1000 enriched genes based on the protein class (**a**) and molecular function (**b**). (**c**) Endocytosis inhibition decreases sensitivity of Panc1 cells to gemcitabine. Panc1 cells were treated for 72 hours with gemcitabine with or without presence of endocytosis inhibitor chlorpromazine. The cells were pre-treated with chlorpromazine for 24 hours. Bars show the average of three experimental replicates ± SD. * and ** represent p-values ≤ 0.05 and ≤0.001 respectively. (**d**) Chlorpromazine inhibits the internalization of fluorescent dextran by endocytosis. Panc1 cells were pre-treated with chlorpromazine for 24 hours and then treated for 3 hours with fluorescent dextran (10 μM). Bars show the average of six experimental replicates ± SD.

Gene composition of the entire library, top 1000 depleted genes and top 1000 enriched genes were extracted employing PANTHER classification system^14^. Figure 4a,b show the gene lists’ compositions based on their protein class and molecular function, respectively.

Comparing protein class of the gene lists, cytoskeletal protein, nucleic acid binding protein and viral protein categories of the enriched genes and transporter category of the depleted genes manifested a significant difference from those of the entire library (Fig. 4a). Regarding molecular function of the genes, signal transduction, structural molecule and transporter categories in top 1000 depleted genes showed significant difference with the entire library (Fig. 4b).

GSEA results, using Gene Ontology All gene sets, revealed that four gene sets including Regulation of Microtubule Based Process, Chromosomal Region, Organelle Assembly and Condensed Chromosome, were significantly enriched in gemcitabine-treated cells (Supplementary Fig. S9).

## Discussion

In order to assess the efficacy of the genome-scale CRISPR/Cas9 knockout experiment GSEA was performed employing Gene Ontology All gene sets from Molecular Signature Database (MSigDB). The result showed that the essential gene sets including Multi Organism Metabolic Process, Ribosomal Subunit and Translational Initiation were depleted in the library of knockout cells after 22 days (Fig. 1c). These results indicate that the genome-scale knockout experiment was performed successfully.

To obtain essential pathways for the survival of Panc1 cells, GSEA employing Hallmark gene sets was performed and revealed that MYC-targets, DNA-repair, G2M-checkpoint and E2F-targets gene sets were essential for Panc1 cells survival (Fig. 1d). These data is consistent with previous reports demonstrating the importance of MYC protein for the survival of Panc1 cells^15,16^. Furthermore, it has been shown that MYC and E2F proteins have transactivation effects on each other^17,18^. These findings further consolidate the previous reports that targeting MYC pathway might be a possible therapeutic approach for treatment of pancreatic cancer.

The human genes that are involved in gemcitabine metabolism can be classified to two categories. Positive gemcitabine modulators, genes that act in accordance with gemcitabine action and negative modulators, genes that act apposed to gemcitabine action and reduce its effectiveness^1^. As an in-silico validation of the gemcitabine screening performed in this study, GSEA was performed using the lists of positive and negative modulators of gemcitabine. As expected, the positive modulators were enriched in gemcitabine-treated cells, while negative modulators did not show any enrichment (Supplementary Fig. S8).

As a result of the gemcitabine screening, *SH3D21* appeared as top gemcitabine sensitizer (Fig. 2b) and *SH3D21*-knockout cells showed more sensitivity to gemcitabine compared to control cells in validation experiments (Fig. 3a,b). Silencing *SH3D21* gene by means of an siRNA increased sensitivity of Panc1 cells to gemcitabine (Fig. 3c,d). Furthermore, re-expression of SH3D21 in *SH3D21*-knockout cells inversed the gemcitabine sensitizing effect of SH3D21 knockout (Fig. 3e,f).

*SH3D21* is expressed in all human tissues and its protein is localized in the nucleus and plasma membrane of the cells^19^. It is not a well-studied gene and in fact an exome array study showing that two *SH3D21* SNPs were associated with resting metabolic rates (RMR)^20^ is the only available report about *SH3D21* function. Employing PICKLES database^21^, we found that SH3D21 gene is essential for the survival of NCIH526 lung cancer cell line, Avana 2018q4 dataset (BF = 9.971, Essentiality Threshold BF = 5). In order to have a deeper insight about the function of SH3D21, Integrative Multi-species Prediction (IMP) server^22^ was used. This server explores predicted biological process that a protein of interest is involved in. The best prediction for SH3D21 biological function was purine ribonucleoside triphosphate catabolic process. Given that gemcitabine needs to be processed to gemcitabine triphosphate inside the cells in order to act as an active drug^23^ this result is strongly in conformance with *SH3D21* gemcitabine sensitizing action. Further studies are required to confirm whether the catabolic activity of SH3D21 on gemcitabine triphosphate is the mechanism by which SH3D21 attenuates gemcitabine effect or other mechanisms are involved.

In previous RNAi-based screenings, *CHEK1*, *VDR* and *RAD17* have been introduced as gemcitabine sensitizers^24–26^. However, these genes did not show up among the top gemcitabine sensitizer candidates in our study. This might be due to the difference in mechanisms of action of knockout (CRISPR/Cas) and knockdown (RNAi) screenings. The former manipulation tool completely abolishes the function of a gene while the latter causes incomplete cessation of the gene’s function. In fact, it has been shown that compared to shRNA, CRISPR/Cas9 system produces more positive hits, 2 to 5 folds, in loss-of-function screenings^27^. Furthermore, as G2M-chekpoint gene set appeared to be an essential gene set for the survival of Panc1 cells (Fig. 1d), with *CHEK1* ranked 729 in the list of essential genes, it is also possible that genes like *CHEK1* and *RAD17*, which are involved in G2M-checkpoint, were observed as top hits in previous studies due to their background effect on the cells survival.

Four Gene Ontology gene sets were enriched in the gemcitabine-treated cells (Supplementary Fig. S9), meaning their leading genes may act as positive regulator of gemcitabine. These gene sets share genes like *KIAA0196*, *CHMP4B*, *SKA3* and *CENPC1* as their top hits that are involved in endosomal transport and cell division^28–31^. The viral protein category that showed a significant increase in top 1000 enriched genes compared to the entire library (Fig. 4a) was composed of three genes, of which two were involved in cell division, *CEP250* and *CEP135*^32^. These results indicate that an intact cell division machinery might be important for mechanism of action of gemcitabine in Panc1 cell line. The importance of the intact cell division machinery is in accordance with previous observation that cell cycle arrest is the major gemcitabine mode of action within ninety-six hours^33^.

The proportion of the proteins with transporter activity was decreased in top 1000 depleted genes compared to those of the entire library (Fig. 4a,b). Of four known gemcitabine transporters only *SLC28A3*^34,35^ was absent among top 1000 depleted genes and present among top 1000 enriched genes. The other known gemcitabine transporters failed to show this pattern. On the other hand, one of the three viral genes that were enriched in gemcitabine-treated cells was *ERVFRD-1*, which is involved in endosomal transport^36^. These results increased the possibility of participation of other transporting molecules/mechanisms, e.g. endocytosis, in gemcitabine uptake by Panc1 cells. To test this hypothesis, we made use of an endocytosis inhibitor, chlorpromazine^37^, and observed that the effect of gemcitabine was attenuated in the presence of chlorpromazine (Fig. 4c). To reassure the inhibitory effect of chlorpromazine on endocytosis, we evaluated the internalization of fluorescent dextran to the Panc1 cells in the presence of chlorpromazine (Fig. 4d). These data confirm that endocytosis is also involved in gemcitabine cellular uptake alongside its known transportation mechanism by transporter proteins.

In brief, we found that *SH3D21* is a gemcitabine sensitizer and may act as a novel therapeutic target to improve gemcitabine efficacy. Also, MYC pathway appeared as an essential pathway for the survival of Panc1 cells in our study and might serve as a target for treatment of pancreatic cancer. Furthermore, we found that endocytosis is involved in gemcitabine cellular uptake, a finding that may help planning new strategies for gemcitabine delivery.

## Methods

### Cell culture and gemcitabine

Human pancreatic cancer Panc1 cells were cultured in RPMI 1640 medium (Sigma R8758) supplemented with 10% fetal bovine serum (FBS) in corning plates. Human embryonic kidney 293FT (HEK293FT) cells were cultured in DMEM medium (Sigma D5796) supplemented with 10% FBS. Gemcitabine was purchased from Sigma (G6423) and was reconstituted in normal saline at the concentration of 10 mg/ml.

### Screening

Panc1 cells were infected with virus particles carrying GeCKO-v2 sgRNA library and were selected by culturing in the medium containing 2 μg/ml puromycin. After seven days of puromycin selection, 20 × 10^6^ cells for library A and 18 × 10^6^ cells for library B were frozen as baseline day 7 samples. Following 22 days of puromycin selection, the Panc1 cells were utilized for screening. Cells of each library, A and B, were split to three parts, one part was frozen as baseline control and two other parts were used for screening by either gemcitabine or vehicle. As baseline controls, 98 × 10^6^ cells for library A and 87 × 10^6^ cells for library B were frozen. For gemcitabine and vehicle control, two sets of 66 × 10^6^ cells and 60 × 10^6^ cells were plated in 15 cm dishes, 3 × 10^6^ cells/dish, for library A and B, respectively. For gemcitabine screening, cells were cultured in the medium containing 0.5 nM gemcitabine (EC20). Equal amount of normal saline was added to the medium of the vehicle control cells. Cells were incubated at the aforementioned conditions for three days and medium was changed every other day. From library A, 98 × 10^6^ cells of either the test or the control condition were frozen. For library B, 87 × 10^6^ cells for each condition were collected and frozen.

### Cell viability assay

Panc1 cells were cultured in 96 well plates, 3000 cells per well in 100 ul of medium. Following 24 hours of incubation, medium was changed and medium containing different concentrations of gemcitabine was added to different wells. The cells were incubated for designated time and the medium was changed every other day. At the desired time point, viability of the cells was measured using CellTiter-Glo kit from Promega (G7572) according to the manufacturer’s guideline.

### *SH3D21*-knockout cells

Lentiviral plasmid vector carrying Cas9 endonuclease and sgRNA targeting *SH3D21* was prepared utilizing lentiCRISPR-v2 vector. Two vectors containing sgRNAs targeting EGFP were also prepared as controls. The spacer sequence used to target *SH3D21* was 5′ CCGCGCTGTGCGCGCCGCCG 3′. Two control spacers targeting EGFP had the following sequences 5′ GAAGTTCGAGGGCGACACCC 3′ and 5′ GGTGAACCGCATCGAGCTGA 3′. Constructed plasmids were utilized for preparation of lentiviral particles. Panc1 cells were transfected by the lentiviral particles at the MOI ~ 0.3 and infected cells were selected with medium containing 2 μg/ml puromycin.

### SURVEYOR assay

For detecting indel mutations at *SH3D21* sgRNA target site SURVEYOR assay was performed utilizing a kit form GeneCopoeia (IC005). The genomic region of interest from *SH3D21* was amplified employing following primers 5′ ATGGGTAAGTGCGGAGGCTTTGAG 3′ and 5′ AGCTGAAGTTCACTTTGCACC 3′. Genomic region containing the sgRNAs target site was PCR-amplified, 800 bp. Control and sample amplicons were mixed and reannealed. The annealing product was digested by T7 endonuclease. Indel mutations of *SH3D21* produce bands with the size of 480 and 320 bp.

### *SH3D21* knockdown

Panc1 cells were reverse transfected with either si-Control or si-*SH3D21* using transfection reagent Lipofectamine RNAiMAX from Thermo Fisher Scientific. Control and *SH3D21* siRNAs were selected from Silencer Select Validated siRNA Library of Thermo Fisher Scientific with catalog numbers of AM4611 and s36203, respectively. Western blot experiment to confirm the activity of si-*SH3D21* was performed using anti-SH3D21 (ab186509) and anti-Tubulin (ab6046) antibodies from abcam.

### SH3D21 re-expression

Panc1 *SH3D21*-KO cells were transfected with either Control or SH3D21 expressing vector (pcDNA 3.1^+^) using transfection reagent Lipofectamine 2000 from Thermo Fisher Scientific. The vector expresses the larger isoform of SH3D21, 84 KD. The medium was changed 24 hours after transfection to gemcitabine containing medium and cell viability assay was performed after 72 hours. Cell viability was measured using CellTiter-Glo kit from Promega (G7572) according to the manufacturer’s guideline. Western blot experiment to confirm the re-expression of SH3D21 was performed using anti-SH3D21 (ab186509) and anti-Tubulin (ab6046) antibodies from abcam, United Kingdom.

### Endocytosis assay

Panc1 cells, 3000 cells per well, were cultured in 96-well plate. After 24 hours the medium was changed to the medium containing chlorpromazine. Following 24 hours of pre-treatment with chlorpromazine, the medium was changed to the medium containing chlorpromazine and gemcitabine. Cell viability was measured after 72 hours of treatment with drugs.

### Fluorescent dextran internalization assay

The cells were cultured in 96 well plates. After 24 hours of pre-treatment with chlorpromazine, the cells were treated with 10 μM fluorescent dextran (Thermo Fisher Scientific D22910) for 3 hours. The cells were washed with culture medium and the internalized fluorescent signal was measured by a fluorescent plate reader.

Further information regarding the methods used in this study can be found in Supplementary Information.

## Supplementary information

Supplementary Information

Dataset 1

Dataset 2

## Data Availability

The datasets generated during and/or analysed during the current study are available from the corresponding authors on reasonable request.
