# Supplementary material for: A genome-scale CRISPR/Cas9 knockout screening reveals SH3D21 as a sensitizer for gemcitabine
Source: Sci Rep. 2019 Dec 16;9:19188. doi: 10.1038/s41598-019-55893-2 (PMC6915784; doi:10.1038/s41598-019-55893-2)
Supplement: Supplementary file 1 — Supplementary Information [file 41598_2019_55893_MOESM1_ESM.pdf]

Supplementary Information

A genome-scale CRISPR/Cas9 knockout screening reveals *SH3D21* as a sensitizer for gemcitabine

Mohammad Masoudi[1,2,3,4], Motoaki Seki[3], Razieh Yazdanparast[4*], Nozomu Yachie[3], Hiroyuki Aburatani[1,2*]

[1]Molecular Biology Department, Graduate School of Medicine, The University of Tokyo, Tokyo 153-8904, Japan

[2]Genome Science Division, Research Center for Advance Science and Technology, The University of Tokyo, Tokyo 153-8904, Japan

[3]Synthetic Biology Division, Research Center for Advance Science and Technology, The University of Tokyo, Tokyo 153-8904, Japan

[4]Molecular Biology Laboratory, Institute of Biochemistry and Biophysics, University of Tehran, Tehran 13145-1384, Iran

[*]To whom correspondence should be addressed.

Plasmid library preparation

The human GeCKO-v2 plasmid library was purchased from Addgene. This library consists of two parts, library A and library B. Lib A contains 65,383 unique sgRNAs and Lib B is composed of 58,028 unique sgRNAs. Each library has 3 sgRNA targeting a gene and in total both library contain 6 sgRNA per gene. In addition, library A includes sgRNAs targeting miRNAs, 4 sgRNA per miRNA. In total both libraries target 19,050 human genes by means of 123,411 unique sgRNAs. To amplify the purchased library, NEB 5 alpha chemically competent *E.Coli* cells were transfected using 500 ng of plasmid DNA of either human GeCKO-v2 library A or B. Following the transfection the cells were incubated at 32°C overnight. The colonies were collected, pooled and the plasmid DNA was extracted using Nucleobond Xtra Plasmid DNA Purification kit (TaKaRa U0410B).

Lentiviral library preparation

For each library, A or B, 90 × 10^6 HEK293FT cells in fifty 10 cm dishes, were cultured and incubated overnight. Next day 150 µg of GeCKO-v2 library plasmids were used for transfection of the cells. The plasmid libraries were mixed with lentiviral packaging plasmids, psPAX2 and PMD2.G, at the ratio of 3:2.25:0.75. The plasmid mixture was used to transfect the cells using lipofectamin 3000 transfection reagent kit (ThermoFisher Scientific). Following an overnight incubation the medium was changed and fresh medium was added to the plates. After incubation for 48 hours the supernatant, containing the virus particles, was collected and stored in -80°C.

Panc1 cells library preparation

The prepared lentiviral libraries were utilized to transduce Panc1 cells at the MOI of ~ 0.3. Trypsinized Panc1 cells (3 × 10^6 cells) were plated in each well of 12 well plate, the virus solution was added to the cells in a medium containing 8 µg/ml polyberen (Sigma), plates were centrifuged for 120 min (2000 RPM, 32°C) and incubated in CO2

incubators for 12 hours. The cells were then trypsinized and plated in 10 cm dishes, 24 hours later the medium was changed to the medium containing 2 μg/ml puromycin.

Next generation sequencing library preparation

Frozen cell pellets were thawed and genomic DNA was extracted using Qiagen Blood & Cell Culture DNA Midi Kit (13343). First PCR reactions were performed using the genomic DNAs as template to amplify regions of the lentiviral vector containing spacer sequences, which had already been incorporated into the host genome. Second PCRs were performed to index the different samples using different index sequences and adhere the illumina P5 and P7 primers to the amplified product (Supplementary Fig. S1). Supposing 6.6 μg of genomic DNA for 10^6 cells 435 and 385 μg of the extracted genomic DNAs were used for library A and library B samples respectively (~ 1000 times of library size). The first PCR reactions were performed utilizing 5 μg of template DNA in 50 ul reaction solutions using following primers and KAPA HiFi hotstart DNA polymerase. Forward primer: 5’-TAACTTACGGAGTCGCTCTACGTCTTGTGGAAAGGACGAAACACCG-3’. Reverse primer: 5’-GGATGGGATTCTTTAGGTCCTGTGTGGGCGATGTGCGCTCTG-3’. After the first PCRs, the entire product for one sample was mixed well and 10 μl of it was used as template for second PCR in four 50 μl PCR reactions. The second PCR was performed utilizing NEB Phusion high fidelity DNA polymerase. Following primers have been used to adhere the P5/P7 Illumina sequencing primers and 9-bases as indexing sequence to each end of the first PCR product. Forward primer: 5’-AATGATACGGCGACCACCGAGATCTACACTCTTTCCCTACACGACGCTCTTCCGATCTNNNNNN [index] TAACTTACGGAGTCGCTCTACG-3’. Reverse primer: 5’-CAAGCAGAAGACGGCATACGAGATCGGTCTCGGCATTCCTGCTGAACCGCTCTTCCGATCTNNNNNN [index] GGATGGGATTCTTTAGGTCCTG-3’. These primers are composed of either P5 (forward) or P7 (reverse) Illumina sequencing

primers followed by 5 random bases, 9 bases as indexing sequence and priming sequence for first PCR product. Products of second PCRs were mixed well and applied to agarose gel electrophoresis (1.5% gel) and the expected bands (395 bp) were cut out of the gels and purified for the next step.

Sequencing

Following NGS library preparation, the libraries were quantified employing KAPA Library Quantification Kit for Illumina sequencing platform (KK4824). After the quantification samples were mixed and 10% Illumina PhiX sequencing control library was added to them. The prepared library mixture was deep sequenced, 100 bases paired-end, by means of Illumina HiSeq 2500 sequencer in high-output mode. The average sequencing depth of 143 for library A and 131 for library B samples were acquired.

Data analysis

Sequencing raw data in FASTQ files were processed and deconvoluted to separate files for each sample using index combinations. Then the reads were processed down to contain spacer sequences merely. The spacer sequences from the source library, human GeCKO-v2, were used to create a Burrows-Wheeler index employing the Bowtie *build-index* function. The sorted reads were aligned to the prepared index, utilizing Bowtie, to extract read number of each unique spacer. The number of the reads for each unique spacer sequence in a given sample was normalized using the following formula:

$$spacer\ normalized\ read = \frac{number\ of\ reads\ of\ the\ spacer}{total\ number\ of\ the\ reads\ of\ the\ sample} \times 10^6 + 1$$

Normalized reads were used to obtain the fold change of individual sgRNAs, in vehicle- or gemcitabine-treated cells, compared to the baseline cells. The RIGER p-value analysis was performed on the acquired fold changes employing GENE-E software and utilizing *weighted sum* method. Gene set enrichment analysis (GSEA) was performed using the software provided by Broad Institute of MIT and Harvard.

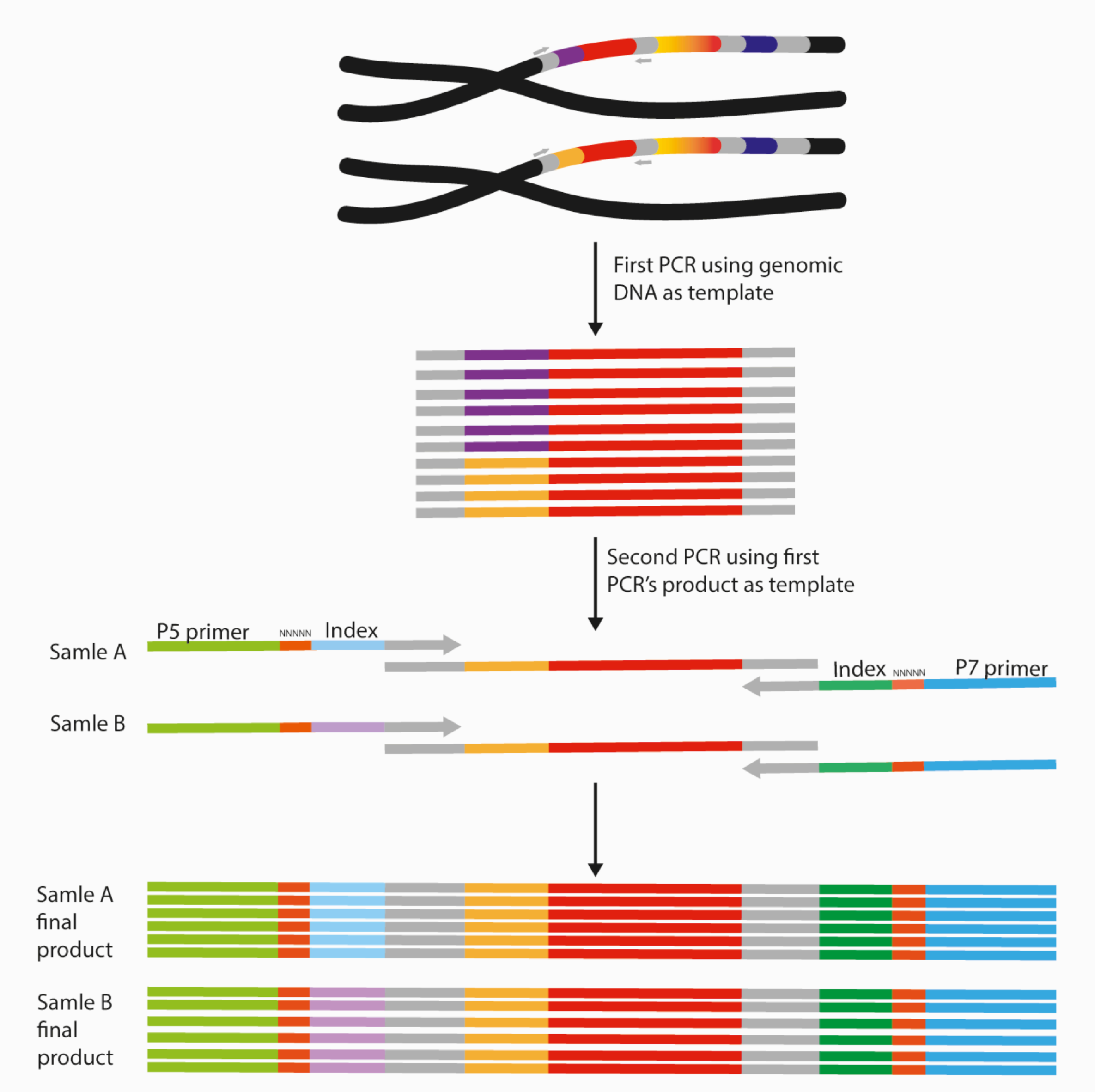

Supplementary Figure S1. Next generation sequencing library preparation overview. The genomic DNA was extracted from the cells and was utilized as the template for the first PCR (18 cycles). The product of the first PCRs for each sample was pooled and 10 µl was used as template for second PCR. The second PCR was performed employing the primers containing either P5 or P7 Illumina sequencing primer, 5 randome bases, 9-bases index and the first PCR primers, forward or reverse respectively. Each screening sample, e.g. baseline, vehicle control or gemcitabine treatment, was indexed by means of a unique index combination.

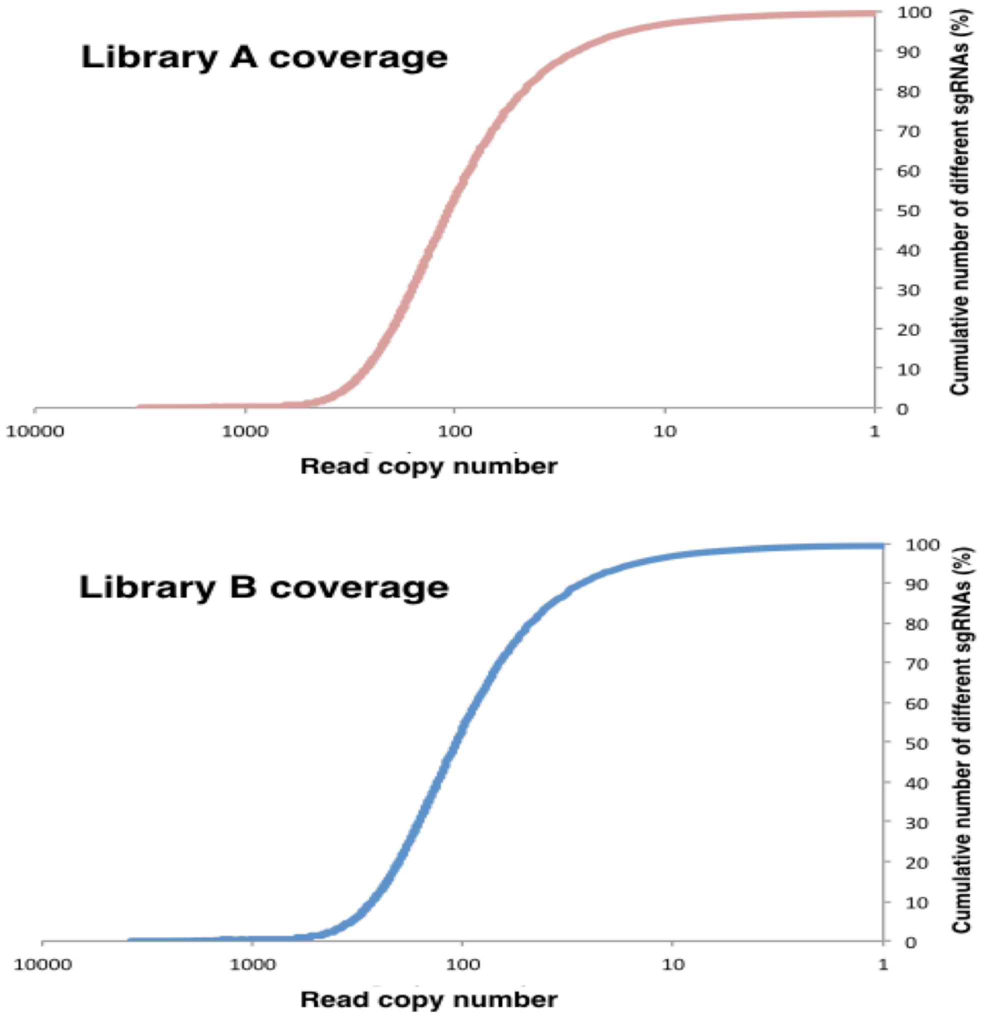

Supplementary Figure S2. sgRNA coverage of the genome-scale knockout experiment 22 days after the start of puromycin selection.

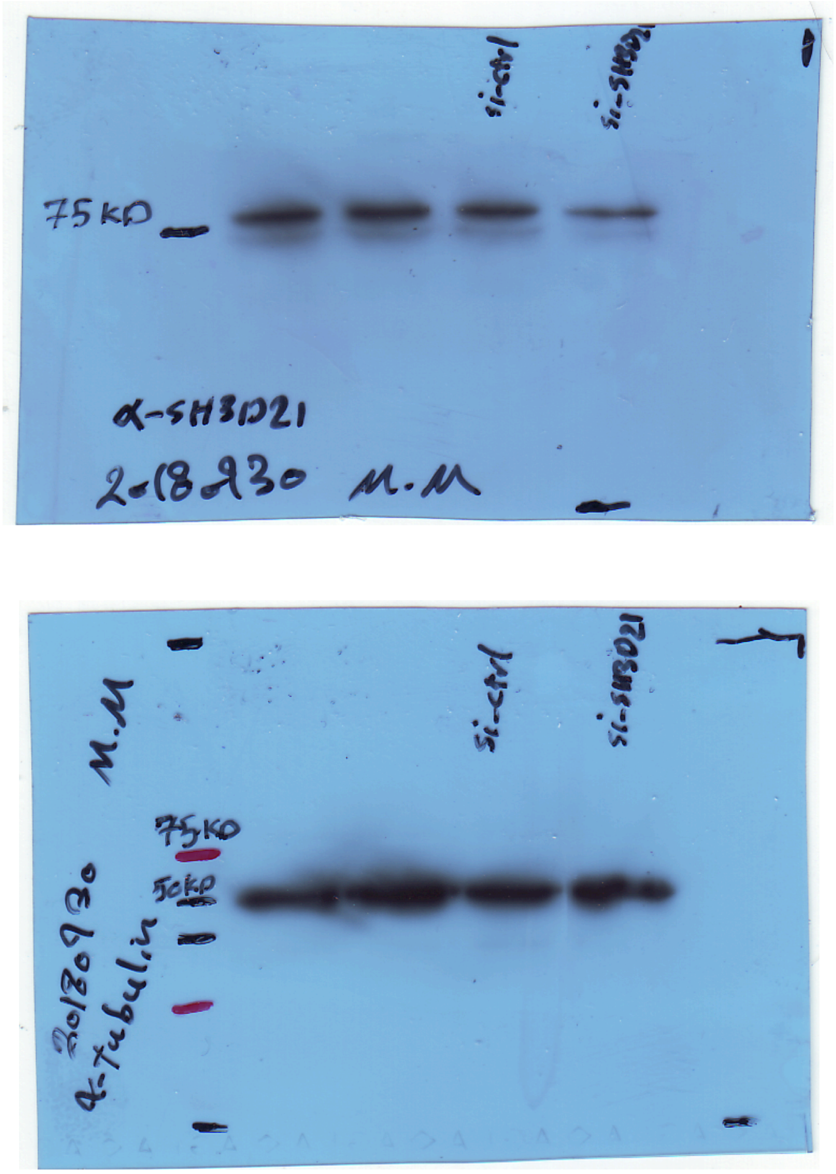

Supplementary Figure S3. Western blot of SH3D21 protein after treatment of Panc1 cells with the si-*SH3D21*. The anti-TUBULIN antibody was used as loading control.

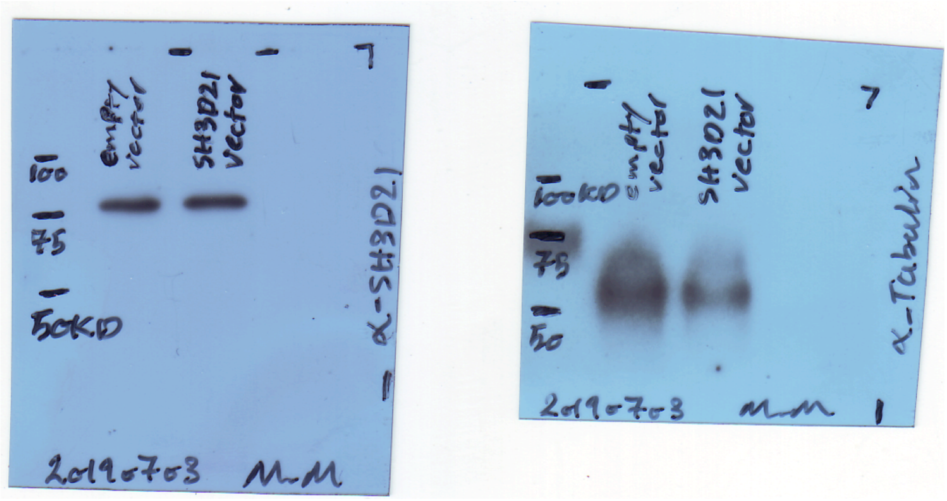

Supplementary Figure S4. Western blot of SH3D21 re-expression in *SH3D21*-knockout cells. The anti-TUBULIN antibody was used as loading control.

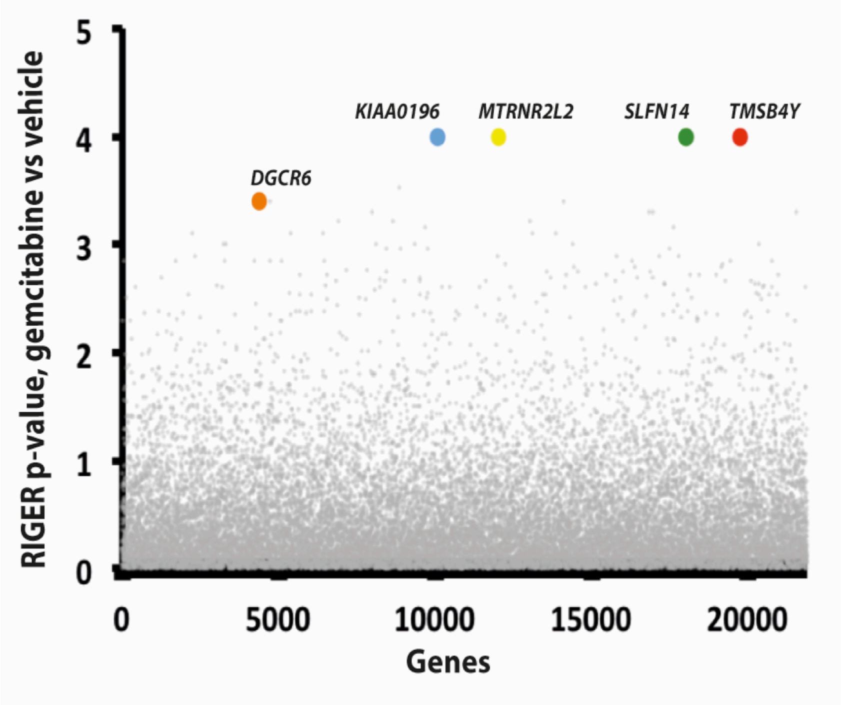

Supplementary Figure S5. RIGER p-value position of the top five enriched genes.

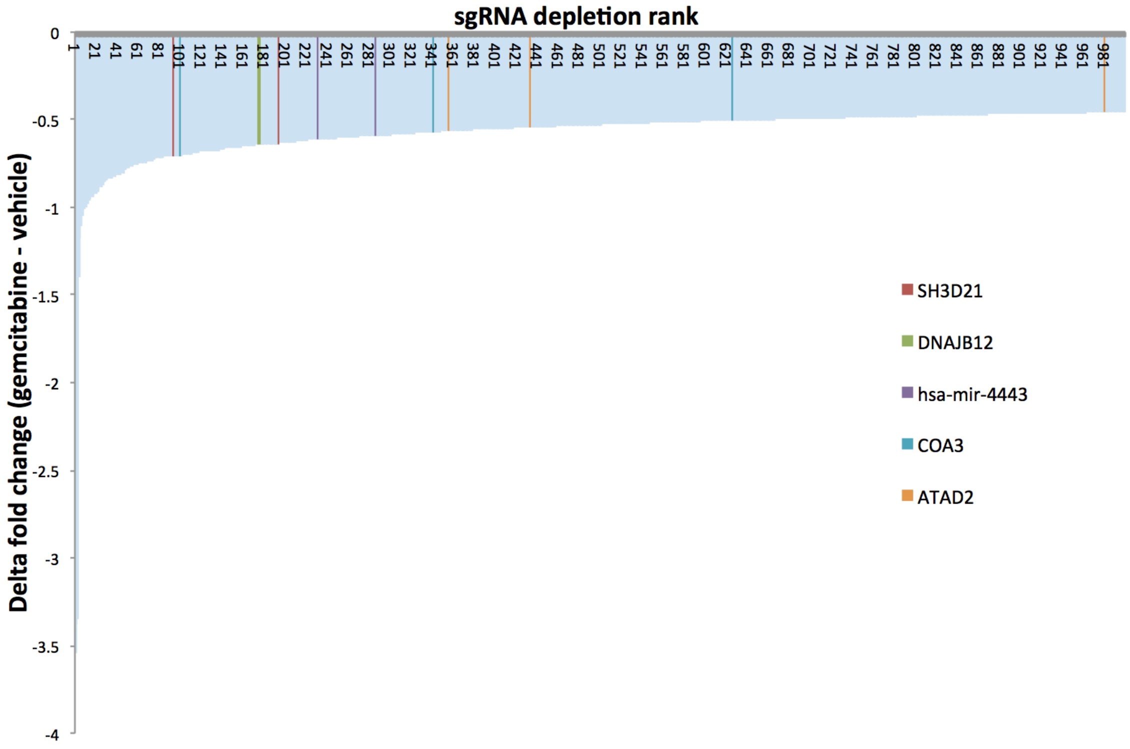

Supplementary Figure S6. Position of the sgRNAs attributed to top five depleted genes among the top 1000 depleted sgRNAs. Note that two DNAJB12 sgRNAs are positioned in tandem.

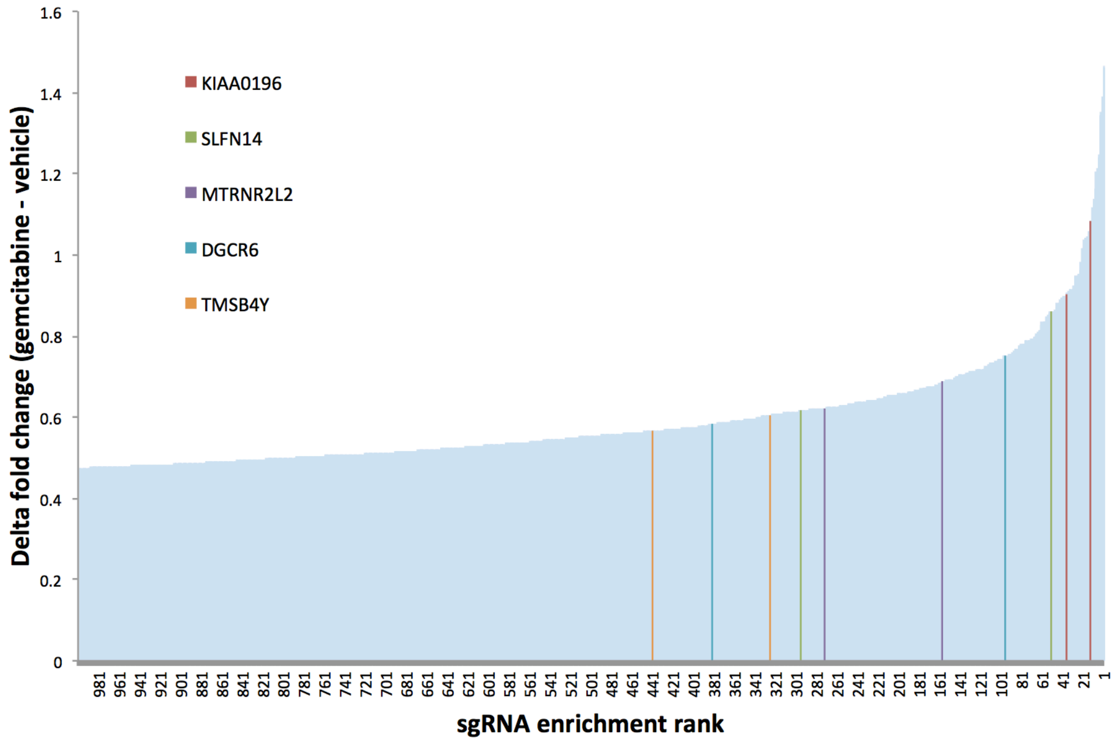

Supplementary Figure S7. Position of the sgRNAs attributed to top five enriched genes among the top 1000 enriched sgRNAs.

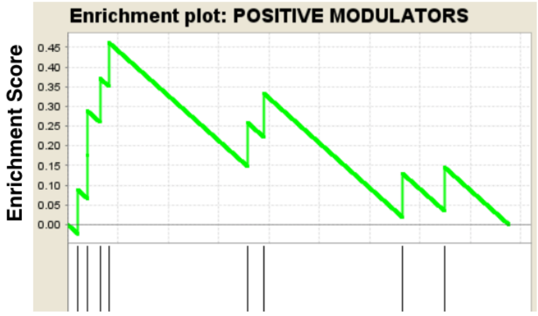

Positive modulators

	Gene name	RANK IN GENE LIST
1	CMPK1	523
2	RRM2	989
3	SLC28A3	998
4	SLC29A1	1610
5	DCK	2049
6	SLC29A2	8929
7	SLC28A1	9732
8	RRM1	16623
9	RRM2B	18702

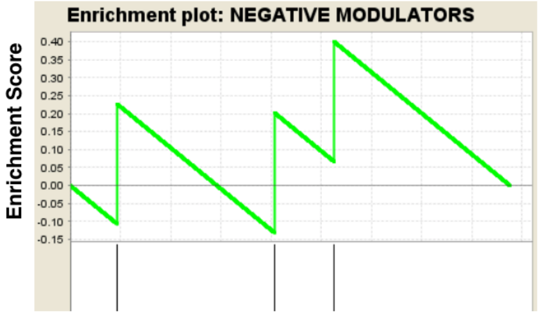

Negative modulators

	Gene name	RANK IN GENE LIST
1	CDA	2327
2	NT5C	10153
3	DCTD	13134

	Gene set	SIZE	NES	NOM p-val	FDR q-val	FWER p-val	RANK AT MAX
1	POSITIVE MODULATORS	9	1.72	0.017	0.031	0.017	2049
2	NEGATIVE MODULATORS	3	0.88	0.593	0.587	0.500	13134

Supplementary Figure S8. GSEA of gemcitabine modulators. Modulators of gemcitabine were classified to positive and negative modulators based on their role in gemcitabine effect and were utilized as gene sets for GSEA.

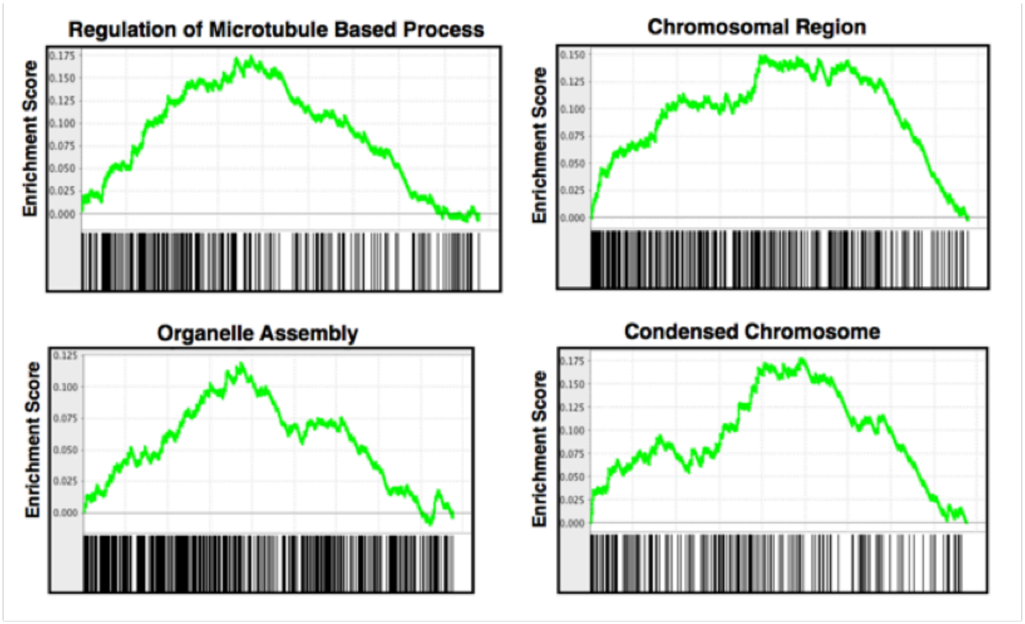

NAME	SIZE	ES	NES	NOM p-val	FDR q-val	FWER p-val	RANK AT MAX
GO_REGULATION_OF_MI CROTUBULE_BASED_PR	234	0.17381458	3.1133738	0	0.003942407	0.003	9334
GO_CHROMOSOMAL_RE GION	316	0.14885034	3.085514	0	0.004612424	0.007	10160
GO_ORGANELLE_ASSEM BLY	471	0.11939375	2.9568727	0	0.006561044	0.015	9342
GO_CONDENSED_CHRO MOSOME	186	0.17783044	2.7582538	0	0.015400141	0.047	12266

Supplementary Figure S9. GSEA of Gene Ontology gene sets in gemcitabine-treated cells. Gene set enrichment analysis using Gene Ontology All gene sets of Molecular Signature Database (MSigDB) with minimum size of 15 and maximum size of 500 for gene sets.
